# Proportion of subjects with psychotic features in bipolar disorder correlated with treatment response by antipsychotics for acute mania

**DOI:** 10.1111/pcn.13460

**Published:** 2022-09-12

**Authors:** Masashi Ikeda, Takeo Saito, Tetsufumi Kanazawa, Taro Kishi, Nakao Iwata

**Affiliations:** ^1^ Department of Psychiatry Fujita Health University School of Medicine Toyoake Japan; ^2^ Department of Neuropsychiatry Osaka Medical College Takatsuki Japan

A recent systematic review and network meta‐analysis provided evidence that several antipsychotics and traditional mood stabilizers (e.g. lithium and valproate) were effective and tolerant for acute mania in bipolar disorder (BD).^1^ Specifically, the results regarding the antipsychotics are convincing for clinicians because such agents can improve (i) elevated/irritable mood and other manic symptoms by their immediate sedative effect and (ii) psychotic symptoms in BD,^1^ although not consistently with a significant correlation.^2^ This raises important speculation on whether antipsychotics should be regarded as “versatile” BD drugs, since they are the “best” treatment option, particularly in cases of difficult diagnosis (e.g. BD with psychotic features: unclear boundary with schizoaffective disorder or schizophrenia [SCZ]).

In the review,^1^ however, BD proportion with psychotic features was not always correlated with treatment response in meta‐regression analysis; however, this analysis had a “power” problem as it was derived based on combining the targeted drugs (antipsychotics + mood stabilizers + other drugs), treating them as “one covariate group.”^1^ Therefore, to clarify this correlation and/or confirm our speculation regarding antipsychotics being a “versatile” drug, we conducted a subgroup‐analysis between such a proportion and improvement rate in acute mania, stratified by drug type, specifically for antipsychotics.

This analysis utilized the studies involved in the previous review^1^ and only used data from double‐blind, randomized placebo‐controlled trials (DBRPCTs) (i) with information on BD proportion with psychotic features, (ii) comparing antipsychotics or traditional mood stabilizers (lithium and valproate) with placebo (outcome: manic symptom improvement, including Young Mania Rating Scale^3^ and Mania Rating Scale^4^: Table S1). Additionally, a meta‐regression analysis was conducted to examine whether the standardized mean difference (SMD) for each drug class was correlated with BD proportion having psychotic features (%). This study was conducted using the Comprehensive Meta‐Analysis software v.3 (Biostat Inc., Englewood, NJ, USA).

Twenty‐eight DBRPCTs (*n* = 8801, 40 comparisons: observational point of main outcome, 3 weeks) were eligible for this analysis (Table S1). For the pooled antipsychotics, we detected a significant correlation between treatment response and percentage of the subjects with psychotic features (beta for SMD = −0.0065, 95% confidence intervals (CIs) = −0.011, −0.0018, *P* = 0.0065: Fig. 1a, Table S2 and Text S1): a higher BD proportion with psychotic features was correlated with improved treatment response. Conversely, no significant trends were observed for mood stabilizers (beta for SMD = −0.0028, 95% CIs = −0.012, 0.0069, *P* = 0.58: Fig. 1b and Table S2).

**Fig. 1:**
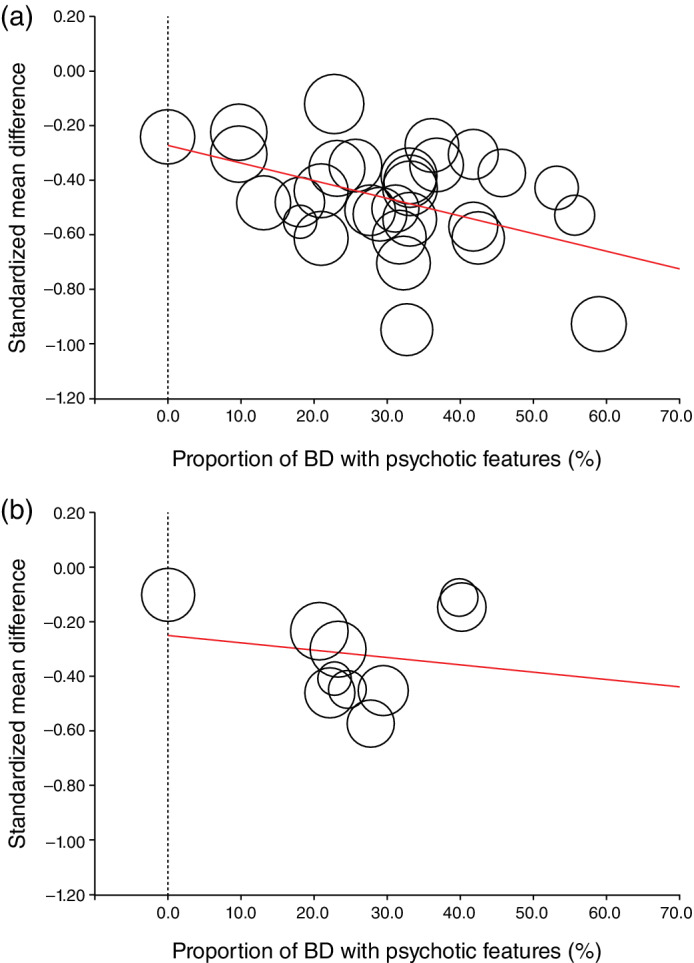
Proportion of bipolar disorder with psychotic features and treatment response by antipsychotics. (a) Based on antipsychotics. (b) Based on mood stabilizers. The X‐axis indicates the proportion of bipolar disorder (BD) with psychotic features. The Y‐axis indicates the standardized mean difference (SMD). Note: We included data from 28 double‐blind, randomized placebo–controlled trials (DBRPCTs), but some of the studies analyzed (i) antipsychotics versus antipsychotics versus placebo (*n* = 8), (ii) antipsychotics versus mood stabilizers versus placebo (*n* = 3) and (3) mood stabilizers versus mood stabilizers versus placebo (*n* = 1). Therefore, 40 comparisons (antipsychotics vs placebo: 30 comparisons, mood stabilizers vs placebo: 10 comparisons) is the final number analyzed in this study.

This subgroup analysis indicated that the proportion of BD with psychotic features was correlated with the treatment response using antipsychotics but not with that using mood stabilizers (as short‐term effect at 3 weeks), although several limitations should be noted (Text S1). This is in line with real‐world clinical practice, where most psychiatrists prescribe antipsychotics for BD patients demonstrating possible psychotic symptoms.

This result partially corresponds to our accompanying research letter, which revealed that BD with psychotic features is correlated with SCZ based on genetic analyses: (i) bipolar I disorder (BD1) in Japan (adhering to “mood” spectrum rather than “psychotic” spectrum) did not show a higher level of shared genetic component with SCZ compared with BD1 in European (EUR) samples (liberal to “psychotic” spectrum), and (ii) proportion of BD1 with psychotic features in Japan (~30%) was much smaller than in EUR (~50%).^5^ [Correction added on 4 Nov 2022, after first online publication: Reference “Saito T, Ikeda M, Terao C et al.” has been inserted as reference 5 and other citations have been renumbered]

It is unclear whether these results were due to sampling bias or cultural “diagnostic attitude” to BD. Nevertheless, based on the results from the current meta‐regression and the accompanying genetic correlation analyses, randomized controlled trials (RCTs) for antipsychotics conducted in Japan and similar areas where psychiatrists adhere to mood symptoms for BD may contain a smaller BD proportion with psychotic features, resulting in worse scores for treatment response using antipsychotics. Simultaneously, RCTs in these areas may prioritize mood stabilizers more than expected or more than antipsychotics.^6^

This speculation is critical in interpreting the clinical study results, including RCTs for BD treatment in the acute^1^ or probably maintenance periods^7^ since antipsychotic effect on BD mania tends to be underestimated in cases of lower BD proportion with psychotic features. However, in most RCTs, the effect of the BD proportion with psychotic features is not emphasized; some RCTs did not mention this information. Therefore, it is recommended that this be described and treated as an important confounding factor in future studies.

## Disclosure statement

The authors have declared that there are no conflicts of interest relating to the subject of this study. Interests from the past 3 years are as follows. Dr. T Kishi has received speaker's honoraria from Sumitomo, Otsuka, Takeda, Eisai, Janssen, and Meiji. Dr. T Kanazawa received research support from Eisai, Sumitomo, Otsuka. Dr. N Iwata received research support or speakers' honoraria from Sumitomo, Eisai, Daiichi Sankyo, Takeda, Meiji, Tanabe‐Mitsubishi, Otsuka, Eli Lilly, Janssen, Viatris. For the remaining authors no conflicts of interest were declared.

## Supporting information


**Table S1** Study characteristics
**Table S2.** The results of meta‐regression analysis
**Text S1**. Heterogeneity and publication bias
