# Supplementary material for: Proportion of subjects with psychotic features in bipolar disorder correlated with treatment response by antipsychotics for acute mania
Source: Psychiatry Clin Neurosci. 2022 Sep 12;76(11):596–8. doi: 10.1111/pcn.13460 (PMC9826388; doi:10.1111/pcn.13460)
Supplement: Supplementary file 1 — Table S1 Study characteristics Table S2. The results of meta‐regression analysis Text S1. Heterogeneity and publication bias [file PCN-76-596-s001.docx]

**Table S1. Study characteristics**

| Study | Treatment (dosing schedule, plasma concentration) | n | Patients with psychotic features in each treatment arm (%) | Total patients | | | | | | | | | | |
| --- | --- | --- | --- | --- | --- | --- | --- | --- | --- | --- | --- | --- | --- | --- |
|  |  |  |  | Patients with psychotic features (%) | Diagnosis | Structured Interview | Severity threshold for mania | Male (%) | Mean age (years) | Patients with rapid cycling (%) | Patients with mixed episode (%) | Observational point of main outcome (week) | Duration (week) | Country |
| Berwaerts 2012^1^ | PAL 3, 6, 12 mg/d (fixed) | 347 | 23.5 | 22.8 | DSM-IV | MINI | YMRS > 20 | 53.5 | 39.5 | 0.00 | 36.8 | 3 | 3 | International |
|  | PLA | 122 | 20.9 |  |  |  |  |  |  |  |  |  |  |  |
| Bowden 1994^2^ | LIT > 1200 mg/d (flexible, < 1.5 mmol/L) | 36 | ni | 24.5 | RDC | RDC | MRS > 14 | 58.1 | 39.6 | ni | ni | 3 | 3 | USA |
|  | VALP > 1000 mg/d (flexible, < 150 µg/mL) | 69 | ni |  |  |  |  |  |  |  |  |  |  |  |
|  | PLA | 74 | ni |  |  |  |  |  |  |  |  |  |  |  |
| Bowden 2005^3^ | LIT initial 900 mg/d (flexible, 0.6-1.4 mEq/L) | 98 | 23.5 | 27.7 | DSM-IV | ni | YMRS > 20 + CGI-BP > 4 | 57.7 | 39.3 | 0.00 | 0.00 | 3 | 12 | International |
|  | QUE 600-800 mg/d (flexible) | 107 | 26.2 |  |  |  |  |  |  |  |  |  |  |  |
|  | PLA | 97 | 33.7 |  |  |  |  |  |  |  |  |  |  |  |
| Bowden 2006^4^ | VALP mean 3060 mg/d (flexible, 85-125 µg/mL) | 187 | 19.3 | 20.6 | DSM-IV-TR | SCID | MRS > 18 | 57.4 | 37.5 | 5.97 | 43.7 | 3 | 3 | USA |
|  | PLA | 177 | 22.0 |  |  |  |  |  |  |  |  |  |  |  |
| Goldsmith 2003^5^ | LIT (flexible, 0.8-1.3 mEq/L) | 36 | ni | 39.7 | DSM-IV | ni | MRS > 18 | 52.8 | 37.9 | ni | ni | 3 | 3 | International |
|  | PLA | 95 | ni |  |  |  |  |  |  |  |  |  |  |  |
| Hirschfeld 2004^6^ | RIS 1-6 mg/d (flexible) | 134 | 40.3 | 42.5 | DSM-IV | SCID | YMRS > 20 + MADRS < 20 | 56.8 | 38.8 | ni | 0.00 | 3 | 3 | USA |
|  | PLA | 125 | 44.8 |  |  |  |  |  |  |  |  |  |  |  |
| Hirschfeld 2010^7^ | VALP mean 2210 mg/d (flexible, mean 83.2 µg/mL) | 147 | ni | 40.1 | DSM-IV | SCID | MRS > 25 | 51.4 | 39.2 | ni | 31.5 | 3 | 3 | USA |
|  | PLA | 78 | ni |  |  |  |  |  |  |  |  |  |  |  |
| Kanba 2014^8^ | ARI 12-24 mg/d (flexible) | 128 | 36.1 | 36.0 | DSM-IV-TR | MINI | YMRS > 20 | 41.3 | 37.7 | 9.31 | 11.3 | 3 | 3 | Asia |
|  | PLA | 130 | 36.0 |  |  |  |  |  |  |  |  |  |  |  |
| Katagiri 2012^9^ | HAL 2.5-10 mg/d (flexible) | 20 | 20.0 | 18.1 | DSM-IV-TR | MINI | YMRS > 20 | 45.3 | 43.4 | 7.24 | 8.14 | 3 | 3 | Japan |
|  | OLA 5-20 mg/d (flexible) | 105 | 16.3 |  |  |  |  |  |  |  |  |  |  |  |
|  | PLA | 99 | 19.6 |  |  |  |  |  |  |  |  |  |  |  |
| Keck 2003 ARI^10^ | ARI 15-30 mg/d (flexible) | 130 | ni | 25.7 | DSM-IV | ni | YMRS > 20 | 43.5 | 40.5 | 23.3 | 32.8 | 3 | 3 | USA |
|  | PLA | 132 | ni |  |  |  |  |  |  |  |  |  |  |  |
| Keck 2003 ZIP^11^ | ZIP 80-160 mg/d (flexible) | 140 | ni | 45.7 | DSM-IV | SCID | MRS > 14 | 54.31 | 38.3 | ni | 35.5 | 3 | 3 | USA and Brazil |
|  | PLA | 70 | ni |  |  |  |  |  |  |  |  |  |  |  |
| Keck 2009^12^ | ARI 15-30 mg/d (flexible) | 155 | 20.0 | 23.1 | DSM-IV-TR | MINI | YMRS > 20 + MADRS < 17 | 51.9 | 39.7 | 0.00 | 39.2 | 3 | 3 | USA |
|  | LIT 900-1500 mg/d (flexible, mean 0.76 mEq/L) | 160 | 24.4 |  |  |  |  |  |  |  |  |  |  |  |
|  | PLA | 165 | 24.8 |  |  |  |  |  |  |  |  |  |  |  |
| Khanna 2005^13^ | RIS 1-6 mg/d (flexible) | 146 | 60 | 59.0 | DSM-IV | ni | YMRS > 20 | 62.0 | 35.1 | 0.00 | 4.49 | 3 | 3 | India |
|  | PLA | 145 | 58 |  |  |  |  |  |  |  |  |  |  |  |
| Kushner 2006 (004)^14^ | LIT 900-1800 mg/d (flexible, 0.8–1.2 mEq/L) | 113 | 27.4 | 26.4 | DSM-IV | SCID | YMRS > 20 | 47.4 | 42.5 | 0.00 | 15.3 | 3 | 3 | International |
|  | PLA | 111 | 24.3 |  |  |  |  |  |  |  |  |  |  |  |
| Kushner 2006 (008)^14^ | LIT 900-1800 mg/d (flexible, 0.8–1.2 mEq/L) | 114 | 23.5 | 22.0 | DSM-IV | SCID | YMRS > 20 | 37.3 | 41.0 | 0.00 | 11.0 | 3 | 3 | International |
|  | PLA | 112 | 20.5 |  |  |  |  |  |  |  |  |  |  |  |
| McIntyre 2005^15^ | HAL 2-8 mg/d (flexible) | 99 | 39.8 | 41.8 | DSM-IV | ni | YMRS > 20 + CGI-BP > 4 | 36.8 | 42.8 | 0.00 | 0.00 | 3 | 12 | International |
|  | QUE 400-800 mg/d (flexible) | 102 | 41.6 |  |  |  |  |  |  |  |  |  |  |  |
|  | PLA | 101 | 44.0 |  |  |  |  |  |  |  |  |  |  |  |
| McIntyre 2009^16^ | ASE 10-20 mg/d (flexible) | 194 | ni | 30.7 | DSM-IV | MINI | YMRS > 20 | 57.4 | 39.4 | 0.00 | 30.7 | 3 | 3 | International |
|  | OLA 5-20 mg/d (flexible) | 190 | ni |  |  |  |  |  |  |  |  |  |  |  |
|  | PLA | 104 | ni |  |  |  |  |  |  |  |  |  |  |  |
| McIntyre 2010^17^ | ASE 10-20 mg/d (flexible) | 185 | ni | 34.3 | DSM-IV | MINI | YMRS > 20 | 52.7 | 38.6 | 0.00 | 31.2 | 3 | 3 | International |
|  | OLA 5-20 mg/d (flexible) | 205 | ni |  |  |  |  |  |  |  |  |  |  |  |
|  | PLA | 98 | ni |  |  |  |  |  |  |  |  |  |  |  |
| Potkin 2005^18^ | ZIP 80-160 mg/d (flexible) | 140 | ni | 31.2 | DSM-IV | SCID | MRS > 14 | 50.7 | 38.9 | ni | 40.5 | 3 | 3 | International |
|  | PLA | 66 | ni |  |  |  |  |  |  |  |  |  |  |  |
| Sachs 2006^19^ | ARI 15-30 mg/d (flexible) | 137 | ni | 13.1 | DSM-IV | ni | YMRS > 20 | 48.5 | 38.8 | 17.7 | 41.5 | 3 | 3 | USA |
|  | PLA | 135 | ni |  |  |  |  |  |  |  |  |  |  |  |
| Sachs 2015^20^ | CARI 3-12 mg/d (flexible) | 158 | 34.8 | 33.0 | DSM-IV-TR | SCID | YMRS > 20 + MADRS < 18 | 64.1 | 36.2 | 0.00 | 9.94 | 3 | 3 | USA and India |
|  | PLA | 154 | 31.2 |  |  |  |  |  |  |  |  |  |  |  |
| Smulevich 2005^21^ | HAL 2-12 mg/d (flexible) | 144 | 34.0 | 33.1 | DSM-IV | ni | YMRS > 20 + MADRS < 20 | 53.0 | 39.8 | 0.00 | 0.00 | 3 | 3 | International |
|  | RIS 1-6 mg/d (flexible) | 154 | 35.1 |  |  |  |  |  |  |  |  |  |  |  |
|  | PLA | 140 | 30.0 |  |  |  |  |  |  |  |  |  |  |  |
| Tohen 1999^22^ | OLA 5-20 mg/d (flexible) | 70 | ni | 53.2 | DSM-IV | SCID | YMRS > 20 | 51.8 | 39.5 | 32.4 | 17.3 | 3 | 3 | USA |
|  | PLA | 69 | ni |  |  |  |  |  |  |  |  |  |  |  |
| Tohen 2000^23^ | OLA 5-20 mg/d (flexible) | 55 | ni | 55.7 | DSM-IV | SCID | YMRS > 20 | 49.57 | 38.7 | 39.1 | 42.6 | 4 | 4 | International |
|  | PLA | 60 | ni |  |  |  |  |  |  |  |  |  |  |  |
| Tohen 2008^24^ | OLA 5-20 mg/d (flexible) | 215 | 0.00 | 0.00 | DSM-IV-TR | SCID | YMRS > 20 + CGI-BP-mania 3 or 4 | 44.5 | 39.6 | 0.00 | 27.1 | 3 | 3 | International |
|  | VALP 500-2500 mg/d (flexible, 50-125 µg/mL) | 201 | 0.00 |  |  |  |  |  |  |  |  |  |  |  |
|  | PLA | 105 | 0.00 |  |  |  |  |  |  |  |  |  |  |  |
| Vieta 2010 ZIP^25^ | HAL 8-30 mg/d (flexible) | 172 | ni | 32.9 | DSM-IV | ni | MRS > 14 | 58.8 | 38.3 | ni | ni | 3 | 3 | International |
|  | ZIP 80-160 mg/d (flexible) | 178 | ni |  |  |  |  |  |  |  |  |  |  |  |
|  | PLA | 88 | ni |  |  |  |  |  |  |  |  |  |  |  |
| Vieta 2010 PAL^26^ | PAL 3-12 mg/d (flexible) | 195 | 18.4 | 21.0 | DSM-IV | ni | YMRS > 20 | 57.6 | 39.2 | 0.00 | 35.2 | 3 | 3 | International |
|  | QUE 400-800 mg/d (flexible) | 193 | 25.0 |  |  |  |  |  |  |  |  |  |  |  |
|  | PLA | 105 | 18.3 |  |  |  |  |  |  |  |  |  |  |  |
| Young 2009^27^ | ARI 15-30 mg/d (flexible) | 167 | 10.2 | 9.69 | DSM-IV-TR | MINI | YMRS > 20 + MADRS < 17 | 44.3 | 40.8 | 0.00 | 18.8 | 3 | 3 | International |
|  | HAL 5-15 mg/d (flexible) | 165 | 7.88 |  |  |  |  |  |  |  |  |  |  |  |
|  | PLA | 153 | 11.1 |  |  |  |  |  |  |  |  |  |  |  |

ARI: aripiprazole, ASE: asenapine, CARI: cariprazine, CGI-BP: Clinical Global Impressions-Bipolar. Version, DSM(-TR); Diagnostic and Statistical Manual of Mental Disorders (-Text Revision), HAL: haloperidol, LIT: lithium, MADRS: Montgomery Åsberg Depression Rating Scale, MINI: Mini International Neuropsychiatric Interview, MRS: Mania Rating Scale (Schedule for Affective Disorders and Schizophrenia), ni. no information, OLA: olanzapine, PAL: paliperidone, PLA: placebo, QUE: quetiapine, RDC: Research Diagnostic Criteria, RIS: risperidone, SCID: Structured Clinical Interview for DSM, VALP: valproate, YMRS: Young Mania Rating Scale, ZIP: ziprasidone

Note: Although we updated our literature search using the same methods as our previous systematic review,^28^ no additional study was required to be included in this study. The risk of bias in each study was also shown in our previous systematic review.^28^ Tohen et al (2008)^24^ targeted the individuals without non-psychotic features; therefore, the proportion of psychotic features were null.

References

1. Berwaerts J, Xu H, Nuamah I, Lim P, Hough D. Evaluation of the efficacy and safety of paliperidone extended-release in the treatment of acute mania: a randomized, double-blind, dose-response study. *J Affect Disord* 2012; **136**: e51-e60.

2. Bowden CL, Brugger AM, Swann AC et al. Efficacy of divalproex vs lithium and placebo in the treatment of mania. The Depakote Mania Study Group. *JAMA* 1994; **271**: 918-24.

3. Bowden CL, Grunze H, Mullen J et al. A randomized, double-blind, placebo-controlled efficacy and safety study of quetiapine or lithium as monotherapy for mania in bipolar disorder. *J Clin Psychiatry* 2005; **66**: 111-21.

4. Bowden CL, Swann AC, Calabrese JR et al. A randomized, placebo-controlled, multicenter study of divalproex sodium extended release in the treatment of acute mania. *J Clin Psychiatry* 2006; **67**: 1501-10.

5. Goldsmith DR, Wagstaff AJ, Ibbotson T, Perry CM. Lamotrigine: a review of its use in bipolar disorder. *Drugs* 2003; **63**: 2029-50.

6. Hirschfeld RM, Keck PE, Jr., Kramer M et al. Rapid antimanic effect of risperidone monotherapy: a 3-week multicenter, double-blind, placebo-controlled trial. *Am J Psychiatry* 2004; **161**: 1057-65.

7. Hirschfeld RM, Bowden CL, Vigna NV, Wozniak P, Collins M. A randomized, placebo-controlled, multicenter study of divalproex sodium extended-release in the acute treatment of mania. *J Clin Psychiatry* 2010; **71**: 426-32.

8. Kanba S, Kawasaki H, Ishigooka J, Sakamoto K, Kinoshita T, Kuroki T. A placebo-controlled, double-blind study of the efficacy and safety of aripiprazole for the treatment of acute manic or mixed episodes in Asian patients with bipolar I disorder (the AMAZE study). *World J Biol Psychiatry* 2014; **15**: 113-21.

9. Katagiri H, Takita Y, Tohen M, Higuchi T, Kanba S, Takahashi M. Efficacy and safety of olanzapine in the treatment of Japanese patients with bipolar I disorder in a current manic or mixed episode: a randomized, double-blind, placebo- and haloperidol-controlled study. *J Affect Disord* 2012; **136**: 476-84.

10. Keck PE, Jr., Marcus R, Tourkodimitris S et al. A placebo-controlled, double-blind study of the efficacy and safety of aripiprazole in patients with acute bipolar mania. *Am J Psychiatry* 2003; **160**: 1651-8.

11. Keck PE, Jr., Versiani M, Potkin S et al. Ziprasidone in the treatment of acute bipolar mania: a three-week, placebo-controlled, double-blind, randomized trial. *Am J Psychiatry* 2003; **160**: 741-8.

12. Keck PE, Orsulak PJ, Cutler AJ et al. Aripiprazole monotherapy in the treatment of acute bipolar I mania: a randomized, double-blind, placebo- and lithium-controlled study. *J Affect Disord* 2009; **112**: 36-49.

13. Khanna S, Vieta E, Lyons B, Grossman F, Eerdekens M, Kramer M. Risperidone in the treatment of acute mania: double-blind, placebo-controlled study. *Br J Psychiatry* 2005; **187**: 229-34.

14. Kushner SF, Khan A, Lane R, Olson WH. Topiramate monotherapy in the management of acute mania: results of four double-blind placebo-controlled trials. *Bipolar Disord* 2006; **8**: 15-27.

15. McIntyre RS, Brecher M, Paulsson B, Huizar K, Mullen J. Quetiapine or haloperidol as monotherapy for bipolar mania--a 12-week, double-blind, randomised, parallel-group, placebo-controlled trial. *Eur Neuropsychopharmacol* 2005; **15**: 573-85.

16. McIntyre RS, Cohen M, Zhao J, Alphs L, Macek TA, Panagides J. A 3-week, randomized, placebo-controlled trial of asenapine in the treatment of acute mania in bipolar mania and mixed states. *Bipolar Disord* 2009; **11**: 673-86.

17. McIntyre RS, Cohen M, Zhao J, Alphs L, Macek TA, Panagides J. Asenapine in the treatment of acute mania in bipolar I disorder: a randomized, double-blind, placebo-controlled trial. *J Affect Disord* 2010; **122**: 27-38.

18. Potkin SG, Keck PE, Jr., Segal S, Ice K, English P. Ziprasidone in acute bipolar mania: a 21-day randomized, double-blind, placebo-controlled replication trial. *J Clin Psychopharmacol* 2005; **25**: 301-10.

19. Sachs G, Sanchez R, Marcus R et al. Aripiprazole in the treatment of acute manic or mixed episodes in patients with bipolar I disorder: a 3-week placebo-controlled study. *J Psychopharmacol* 2006; **20**: 536-46.

20. Sachs GS, Greenberg WM, Starace A et al. Cariprazine in the treatment of acute mania in bipolar I disorder: a double-blind, placebo-controlled, phase III trial. *J Affect Disord* 2015; **174**: 296-302.

21. Smulevich AB, Khanna S, Eerdekens M, Karcher K, Kramer M, Grossman F. Acute and continuation risperidone monotherapy in bipolar mania: a 3-week placebo-controlled trial followed by a 9-week double-blind trial of risperidone and haloperidol. *Eur Neuropsychopharmacol* 2005; **15**: 75-84.

22. Tohen M, Sanger TM, McElroy SL et al. Olanzapine versus placebo in the treatment of acute mania. Olanzapine HGEH Study Group. *Am J Psychiatry* 1999; **156**: 702-9.

23. Tohen M, Jacobs TG, Grundy SL et al. Efficacy of olanzapine in acute bipolar mania: a double-blind, placebo-controlled study. The Olanzipine HGGW Study Group. *Arch Gen Psychiatry* 2000; **57**: 841-9.

24. Tohen M, Vieta E, Goodwin GM et al. Olanzapine versus divalproex versus placebo in the treatment of mild to moderate mania: a randomized, 12-week, double-blind study. *J Clin Psychiatry* 2008; **69**: 1776-89.

25. Vieta E, Ramey T, Keller D, English PA, Loebel AD, Miceli J. Ziprasidone in the treatment of acute mania: a 12-week, placebo-controlled, haloperidol-referenced study. *J Psychopharmacol* 2010; **24**: 547-58.

26. Vieta E, Nuamah IF, Lim P et al. A randomized, placebo- and active-controlled study of paliperidone extended release for the treatment of acute manic and mixed episodes of bipolar I disorder. *Bipolar Disord* 2010; **12**: 230-43.

27. Young AH, Oren DA, Lowy A et al. Aripiprazole monotherapy in acute mania: 12-week randomised placebo- and haloperidol-controlled study. *Br J Psychiatry* 2009; **194**: 40-8.

28. Kishi T, Ikuta T, Matsuda Y et al. Pharmacological treatment for bipolar mania: a systematic review and network meta-analysis of double-blind randomized controlled trials. *Mol Psychiatry* 2021.

**Table S2. The results of meta-regression analysis**

Pooled antipsychotics *vs*. placebo

| **Covariate** | **Coefficient** | **Standard error** | **95% CI** | **Z-value** | **P value** |
| --- | --- | --- | --- | --- | --- |
| Intercept | –0.270 | 0.0769 | –0.420, –0.119 | –3.51 | 4.51 × 10^–4^ |
| Percent of the subjects with psychotic features | –0.00647 | 0.00238 | –0.0111, –0.00181 | –2.72 | 6.52 × 10^–3^ |

Pooled traditional mood stabilizers (lithium and valproate) *vs*. placebo

| **Covariate** | **Coefficient** | **Standard error** | **95% CI** | **Z-value** | **P value** |
| --- | --- | --- | --- | --- | --- |
| Intercept | –0.244 | 0.129 | –0.496, 0.00888 | –1.89 | 5.86 × 10^–2^ |
| Percent of the subjects with psychotic features | –0.00276 | 0.00493 | –0.0124, 0.00689 | –0.560 | 5.75 × 10^–1^ |

**Supplementary Text**

Heterogeneity and publication bias

We assessed the heterogeneity and publication bias of the pooled samples in the current study:

Pooled antipsychotics outperformed placebo in the improvement of mania rating scale scores (SMD = −0.421, 95% CI = −0.489, −0.352, p < 0.0001, I^2^ = 63.4%; Egger's regression test: p = 0.0664). The results from the I^2^ and Egger’s test indicated that there was no publication bias, although some heterogeneity existed. However, it is of note that the meta-regression analysis decreased I^2^ value to 53.3%, possibly supporting for the significant correlation found between the proportion of BD subjects with psychotic features and treatment response (SMD).

Pooled mood stabilizers also outperformed placebo in the improvement of mania rating scale scores (SMD = −0.380, 95% CI = −0.502, −0.258, p < 0.0001, I^2^ = 58.1%; Egger's regression test: p = 0.274). Again, our meta-regression analysis decreased I^2^ value to 24.5%.

Limitations

We must note that there are several limitations in the interpretation of our current results. First, due to the insufficient power of the “comparisons” between mood stabilizers and placebo, for which we included 10 comparisons, we cannot deny the possibility of false negative results for this analysis. Second, we do not have sufficient data regarding the long-term effects of antipsychotics for mania with psychotic features, although we speculate similar trends for the current results (short-term effect: <3 weeks) will be obtained. In addition, we cannot provide any clear evidence whether a combination of mood stabilizers + antipsychotics will enhance the improvement of psychotic mania. Therefore, further clinical studies in various treatment courses and/or comparisons will be required to obtain conclusive results.

Authorship

MI, TS, TKishi and NI contributed to the conception and study design. MI, TS, TKanazawa, TKishi and NI provide substantial contributions to analysis and interpretation of clinical data. MI, TS and TKishi wrote the first draft of the article. All authors have contributed to and approved the final version of the manuscript.
